# Vinpocetine alleviated alveolar epithelial cells injury in experimental pulmonary fibrosis by targeting PPAR-γ/NLRP3/NF-κB and TGF-β1/Smad2/3 pathways

**DOI:** 10.1038/s41598-024-61269-y

**Published:** 2024-05-15

**Authors:** Zeena A. Hussein, Ahmed R. Abu-Raghif, Nibras J. Tahseen, Khalid A. Rashed, Nada S. Shaker, Hayder Adnan Fawzi

**Affiliations:** 1 Department of Pharmacology, College of Medicine, Al-Nahrain University, Baghdad, Iraq; 2 Department of Pharmacology and Toxicology, College of Pharmacy, Al-Nahrain University, Baghdad, Iraq; 3 Department of Pharmacology and Toxicology, College of Pharmacy, Al-Bayan University, Baghdad, Iraq; 4 Yeovil District Hospital, Somerset, UK; 5 Department of Pharmacology and Toxicology, College of Pharmacy, Mustansiriyah University, Baghdad, Iraq; 6 Department of Pharmacy, Al-Mustafa University College, Baghdad, Iraq

**Keywords:** Bleomycin-induced pulmonary fibrosis, MRC-5 cell line, NLRP3 inflammasome, PPAR-γ, TGF-β1/Smad2/3 pathway, Vinpocetine, Clinical pharmacology, Pharmacology

## Abstract

This study aimed to investigate the potential anti-fibrotic activity of vinpocetine in an experimental model of pulmonary fibrosis by bleomycin and in the MRC-5 cell line. Pulmonary fibrosis was induced in BALB/c mice by oropharyngeal aspiration of a single dose of bleomycin (5 mg/kg). The remaining induced animals received a daily dose of pirfenidone (as a standard anti-fibrotic drug) (300 mg/kg/PO) and vinpocetine (20 mg/kg/PO) on day 7 of the induction till the end of the experiment (day 21). The results of the experiment revealed that vinpocetine managed to alleviate the fibrotic endpoints by statistically improving (P ≤ 0.05) the weight index, histopathological score, reduced expression of fibrotic-related proteins in immune-stained lung sections, as well as fibrotic markers measured in serum samples. It also alleviated tissue levels of oxidative stress and inflammatory and pro-fibrotic mediators significantly elevated in bleomycin-only induced animals (P ≤ 0.05). Vinpocetine managed to express a remarkable attenuating effect in pulmonary fibrosis both in vivo and in vitro either directly by interfering with the classical TGF-β1/Smad2/3 signaling pathway or indirectly by upregulating the expression of Nrf2 enhancing the antioxidant system, activating PPAR-γ and downregulating the NLRP3/NF-κB pathway making it a candidate for further clinical investigation in cases of pulmonary fibrosis.

## Introduction

Pulmonary fibrosis (PF) is a chronic progressive lung disorder characterized by thickened fibrotic alveolar walls leading to impaired gas transfer, restricted ventilatory patterns, and, as a result, respiratory failure^1^. PF is a heterogeneous disease characterized by a distinct pattern of tissue pathology. It comprises a large number of chronic respiratory pathologies accompanied by connective tissue growth in various lung compartments, among which interstitial lung disease (ILD) and idiopathic pulmonary fibrosis (IPF) are the most severe and irreversible ones with progressive fibrosing of the lung parenchyma^2^. Generally, PF development is often preceded by acute lung inflammation caused by viral and bacterial infections, ionizing radiation, chemotherapy, air irritants, and pollutants, which were not resolved in time and resulted in the deposition of fibrotic tissue in the lungs and respiratory dysfunction^3^. It should be noted that the etiology of IPF is unknown, and the causal agent or specific association has not been determined. Still, among the many intrinsic and extrinsic risk factors, viral infections, gastroesophageal reflux disease (GERD), and micro-aspiration-related genetic predisposition are distinguished^4^.

Repairing injured tissues is a necessary biological feature that enables the orderly regeneration of defective or diseased cells. Nevertheless, there are cases where this mechanism appears poorly developed^5^. In that case, it may result in the formation of a perpetual fibrotic “scar” at the source of tissue damage, which is defined by an irregular aggregation of extracellular matrix (ECM) constituents (e.g., fibronectin, interstitial collagens, hyaluronic acid, and proteoglycans)^6^. Consequently, fibrogenesis is often considered an irrational wound-repairing method^7^. PF is characterized as a dysregulated tissue injury/repair process resulting from continuous interstitial tissue injury and the persistent presence of harmful stimuli, collagen deposition, and excessive accumulation of the ECM, eventually resulting in the decline of pulmonary function^8^. Although the detailed mechanism of PF has yet to be disclosed, previous studies have reported the roles of cytokines on pulmonary fibrogenesis, such as interleukin-6 (IL-6), IL-13, and transforming growth factor-beta (TGF-β1). TGF-β1 is one of the most crucial pro-fibrotic factors among these cytokines in tissue repair and fibrogenesis^8^.

Many cytokines are involved in the development of PF, and TGF-β1 is the central regulator in the progression of PF^9^. It regulates the pathogenesis of PF through some signaling pathways, including Smad, MAPK, Wnt, and ERK pathways^9,10^. During the injury and acute phase, TGF-β1 is secreted by alveolar epithelial cells (AECs), which influence the release of necessary cytokines involved in inflammation and initiation of fibrosis (IL-1β, TNF-α, IL-6, etc.)^11^. On the other hand, during the fibrosis stage, there is increased synthesis of TGF-β1 by multiple cell types, including (macrophages, platelets, and T cells)^12^. TGF-β1, through Smad-dependent signaling, stimulates the synthesis of ECM components and regulates fibroblast into myofibroblast differentiation^13^. Myofibroblasts can produce collagen, such as Col 1A1 and Col 3A1, and fibronectin, which constitute the main substances of the extracellular matrix^14^. The overproduction of collagen and the ECM disturbs normal physiological repair in lung tissue^15^. Inhibiting TGF-β signaling could potentially treat pulmonary fibrosis^16^.

This study will focus on some molecular targets, including Nuclear factor erythroid 2-related factor 2 (Nrf2), a regulatory nuclear factor that influences the activity and cellular response of the antioxidant system^17^, NF-κB, and the inflammasome NLRP3 which are closely related to one another and influence the level of inflammatory and pro-fibrotic cytokines in the microenvironment as well as the apoptosis and senescence of alveolar epithelial cells especially for NF-κB^18^. As for peroxisome proliferator-activated receptor gamma (PPAR-γ), studies have suggested its important role in modulating the inflammatory and fibrotic responses in the lung^19^. PPAR-γ is a nuclear receptor that regulates the expression of genes involved in lipid metabolism, glucose homeostasis, and inflammation. PPAR-γ is expressed in many lung cells, including fibroblasts, ciliated airway epithelial cells, alveolar type II pneumocytes, alveolar macrophages, T lymphocytes, and airway smooth muscle cells^20^. PPAR-γ ligands upregulate transcription of genes that oppose myofibroblast differentiation by interfering with TGF-β1 signaling via the Smad pathway in some cell types and suppress the production of pro-inflammatory cytokines and chemokines by various cell types by negatively affecting the nuclear transcriptional factor NF-κB^21^; in addition to measuring collagen, collagen fragments, and α-SMA as distinct biomarkers for establishing fibrosis. Krebs von den Lungens-6 (KL-6) is considered a predictive biomarker of the severity of fibrosis. KL-6 is a high-molecular-weight glycoprotein encoded by the MUC1 gene (Mucin 1 gene) and distributed mainly on the cell surface of type II alveolar epithelial cells (AECs)^22^. As a result of epithelial injury, the disulfide bond will be disrupted, and KL-6 will eventually be diffused into the blood circulation^23^.

Vinpocetine (VCN) is a semi-synthetic derivative of the vinca alkaloid (vincamine), an alkaloid extracted from the leaves of the periwinkle plant, Vinca minor, and the plant belongs to the Apocynaceae family. Chemically, it is known as ethyl apovincaminate. It is commonly sold as a prescription medication in many European countries and Japan under the brand name Cavinton^®^^24^. VCN is mainly an inhibitor of the phosphodiesterase-1 enzyme (the enzyme responsible for the degradation of cGMP and cAMP), with a high affinity towards (PDE1A/1B) isozymes^25^. It also acts as a blocker for voltage-dependent Na + channels^26^. VCN was reported to be an inhibitor of IκB kinase (IKK). IKK plays a critical role in mediating cellular inflammatory responses. NF-κB is liberated due to IκB degradation and then enters the nucleus to activate the transcription of inflammatory molecules. Therefore, by inhibiting IKK activity, VCN acts as a novel, potent anti-inflammatory agent^24,27^. VCN has been mainly implicated in clinical and preclinical studies for the treatment of several Central Nervous System (CNS) disorders, such as cerebral ischemia, epilepsy, Alzheimer’s disease, and Parkinson’s disease^28^. In addition to this, VCN has been reported to show potential in the treatment of several inflammatory disorders, stress, glaucoma, and gastric disorders^29^; in addition to these activities of VCN, a study published in 2021 by Choi et al. about the anti-inflammatory effect of vinpocetine in a model of induced allergic asthma in mice made this agent interesting to investigate its activity in pulmonary fibrosis further^30^.

The present study was designed to investigate the attenuating effect of VCN in bleomycin-induced pulmonary fibrosis in BALB/c mice (in vivo) and human fetal lung fibroblasts (MRC-5) cell line (in vitro) by examining its effect on important cellular and molecular targets (Nrf2, NLRP3, NF-κB, PPAR-γ, fibronectin, vimentin, Smad, and α-SMA) involved in pulmonary fibrosis.

## Materials and methods

### Chemicals and reagents

All chemicals included in the experiment were purchased from different sources: bleomycin sulfate, carboxymethyl cellulose, pirfenidone, and VCN were purchased from (HyperChem, China). Ketamine HCl (Hameln, Germany). Xylazine HCl (XYL-M2, VMD^®^ Livestock Pharma, Belgium). ketamine (Ketamine 10%, Alfasan Nederland BV, Holand). All mouse ELISA kits were purchased from (MyBioSource, USA). TRIzol reagent and tissue lysis reagent were purchased from (Thermo Fisher Scientific, USA). RT-qPCR primers (Macrogen, Korea). Immunohistochemistry antibodies, diluent, HRP-conjugated IgG, and DAB chromogen were purchased from (Biorbyt, UK).

### Ethical statement

Animal handling, experimental procedures, and euthanasia were carried out according to the guidelines for animal care of the National Institute of Health and the American Veterinarian Medical Association (AVMA) 2020^31^. An approval statement was granted by the Animal Ethical Committee of the College of Pharmacy/Al-Nahrain University (No.: nah.col.pha.19, date: 24^th^ October 2022). The authors complied with the ARRIVE 2.0 guidelines^32^.

### Experimental animals and housing

The study was conducted in the animal house of the College of Pharmacy / Al-Nahrain University. BALB/c albino male mice aged (10–12) weeks were purchased from the Al-Rhazi Center / Ministry of Industry and Minerals. Animals were housed in clean and sterile conditions with (23 ± 2) °C temperature and humidity around 45%, and a 12-h light/night cycle and left to acclimatize to the new environment for one week with free access to water, food, and ad libitum. Cage bedding was changed every other day with new sterile bedding throughout the entire experiment.

### Preparation of reagents

Bleomycin solution was prepared by dissolving the weighed powder of bleomycin sulfate in 10 ml of 0.9% normal saline to achieve a final concentration of (1mg/ml). Carboxymethyl cellulose (CMC) stock solution was prepared by preheating distilled water (DW) at 54–56 °C and then adding the required amount of CMC gradually with constant stirring until complete dissolution; this should be done using a water bath to keep the temperature constant. An oral solution of pirfenidone (PRD) was prepared by dissolving the weighed amount in 10 ml of previously prepared solution of 0.5% CMC^33^ to achieve suspension with the aid of vortex and heating at 37 °C to a final concentration of (25mg/ml). VCN oral solution was prepared similarly, except it had to be ground into fine powder before preparing the solution with a concentration of (2 mg/ml).

### Study design and animal grouping

BALB/c albino male mice were used as the suitable animal strain for the experiment. Forty mice aged (10–12) weeks weighing (20–25) grams were randomly allocated into four groups (using block randomization) with ten animals in each group (n = 10). Group 1 (Normal control)/animal groups received only 0.9% normal saline through oropharyngeal (OP) aspiration in a volume equivalent to that of the induced group based on body weight. Group 2 (Induced/BLM)/animals received only a single dose of bleomycin (BLM) (5 mg/kg dissolved in 0.9% NS) through oropharyngeal aspiration^34^. Group 3 (BLM + PRD)/animals received a single dose of bleomycin/OP and a daily dose of pirfenidone (PRD) (300 mg/kg/day, PO), which served as the standard treatment of pulmonary fibrosis^34^. Group 4 (BLM + VCN)/animals received a single dose of bleomycin/OP and a daily dose of VCN (20 mg/kg/day, PO)^35^. Animal groups (3 and 4) were treated with PRD and VCN agents with daily oral dosages from day 7 of the induction till day 21^33^.

**Figure 1:**
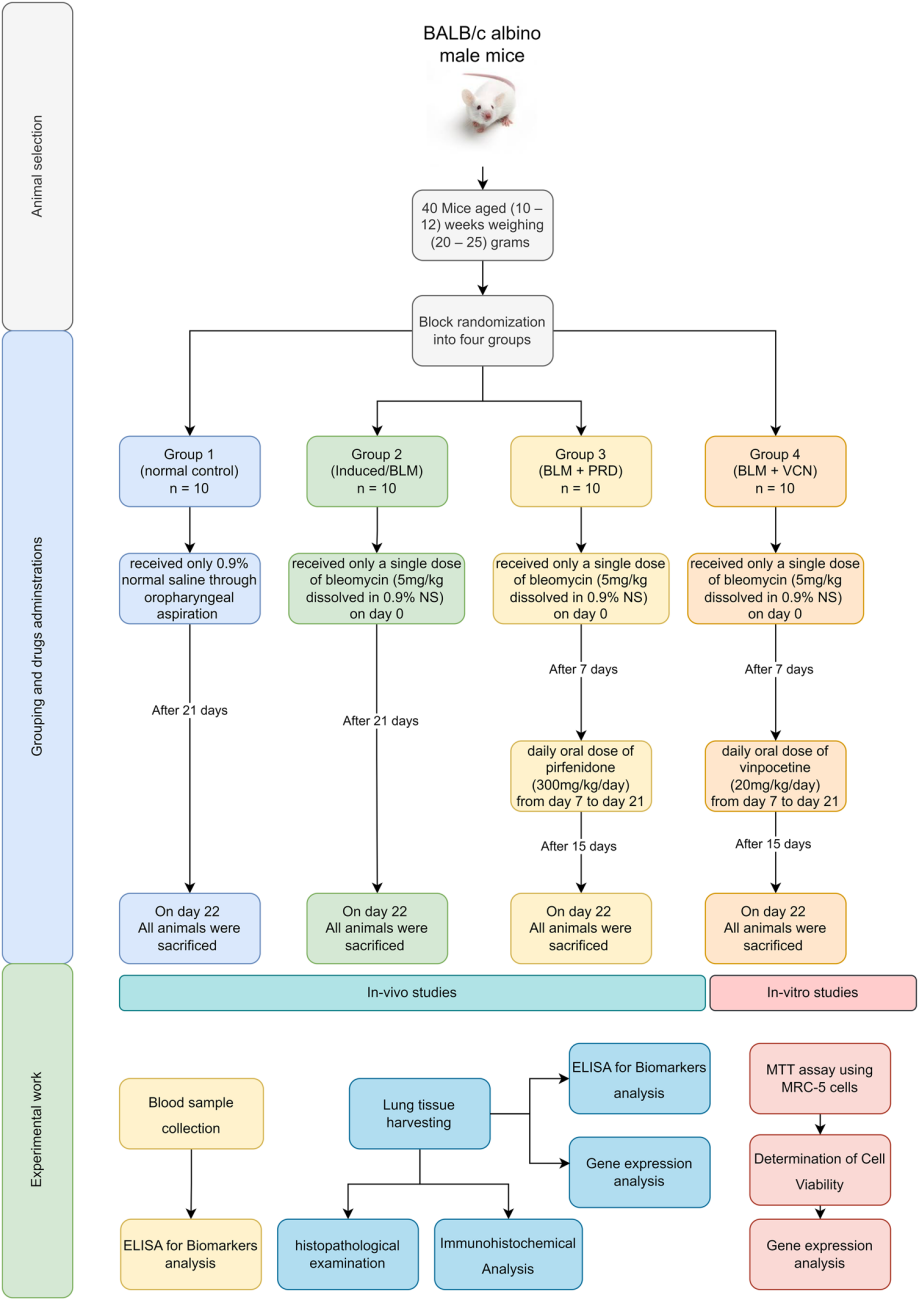
Flow chart of the study.

All animals were sacrificed on day 22 of the experiment; all mice were anesthetized intraperitoneally (IP) with 80 mg/kg of ketamine and 10 mg/kg of xylazine; following total anesthesia, all mice were terminated by exsanguination (cardiac puncture; a procedure suitable for tissue harvest and conservation^31,36^, blood and tissue samples were collected for further analysis. The flow chart of the experiment is illustrated in Fig. 1.

### Induction of pulmonary fibrosis

Animals were anesthetized using (50mg/kg IP ketamine HCl^37^ and 5 mg/kg IP xylazine^38^), and a bleomycin solution was administered through endotracheal (oropharyngeal) catheter intubation of the lung with the assistance of Auroscope (to aid in the visualization of the vocal cords and laryngeal opening). Mice were positioned on the intubation platform, hanging them by their incisors placed on the wire. The tongue was pulled out and held with forceps, and the bleomycin solution was administered through the intubated catheter and aspirated into the lung spontaneously. Mice were monitored in cages until they were fully recovered^39^.

## Samples collection

Blood samples were collected from the animals through heart puncture^40^. Blood was collected in a clot activator tube and centrifuged at 5000 rpm for 10 min to separate serum. The serum was collected in 2 ml Eppendorf tubes and stored at − 20 °C until the analysis day.

After the animals were completely sacrificed, they were dissected, and the entire lung was excised and washed with 0.9% normal saline, then dried from the excess fluids on filter paper^41^. The lung of each animal was weighed and then cut into two parts (left and right) for further analysis. One part of the lung was kept in 10% formalin for further histopathological examination and immunohistochemistry^42^. A small part of the remaining lung, about (30–50) mg, was stored in a TRIzol^®^ reagent for RNA extraction and gene expression analysis. In contrast, most of the remaining part was used to homogenate tissue and further analyze certain tissue biomarkers.

## Lung weight index

Body weight was measured immediately at the end of the experiment (day 22) before sacrificing the animal; body weight was expressed in grams. After sacrificing the animal, the lung tissue was excised, washed, dried, and weighed to calculate the lung weight index by the following formula^14^:

$$Lung\, Weight\, Index\, =\, \frac{Lung\, Wt.\,\left(mg\right)}{Final\, body \,Wt.\, (gm)} \times 100$$

### Enzyme-linked immunosorbent assay (ELISA) technique

Lung tissue homogenate was prepared by adding 1 ml of cold homogenization buffer to 100 mg of lung tissue, followed by homogenization with a Polytron homogenizer using an ice bucket to keep the sample cold^14^. Homogenization buffer contained (1 mM PMSF, one mcg/mL pepstatin A, one mcg/mL aprotinin, and one mcg/mL leupeptin in phosphate-buffered saline, pH 7.2, with 0.05% sodium azide and 0.5% Triton X 100). Homogenate samples were then centrifuged at 12,000 rpm for 10 min. Then, the supernatant was collected in Eppendorf tubes for later analysis^43^. Tissue homogenate samples were stored at – 20 °C until the day of analysis.

Biomarkers of serum and lung tissue homogenate samples were assessed using the ELISA technique. Frozen samples were left to thaw at room temperature, and the biomarkers required assessment for the study include (KL-6, Col IVα1, and Col IVα3) for serum samples (Hydroxyproline, TGF-β1, IL-1β, IL-6, IL-13, TNF-α, MDA, GSH, SOD, and GPx) for lung tissue homogenate. Levels of each of the mentioned biomarkers were evaluated according to the manufacturer’s kit, and the absorbance was measured using a microplate reader (HumaReader^®^, USA) at a wavelength of 450 nm. The concentration of each sample was determined according to the standard curve supplied by each manufacturing kit.

### Histopathological examination and scoring

The lungs were cut into two parts; the left part was fixed in 10% buffered formalin for 48 h at room temperature and then processed to obtain the final tissue-paraffin-embedded blocks. Sections (thickness, five μm) would be stained with hematoxylin and eosin (H&E) and Masson’s trichrome (TM). Two pathologists blinded to the animal groups of the experiment assessed histopathological changes, including alveolitis and fibrosis scores^16^. The alveolitis and fibrosis scores were evaluated according to the criteria developed by Szapiel^44^ and Ashcroft^45^, as in Tables 1 and 2.

**Table 1:** Criteria for scoring alveolitis (inflammation)^44,46^.

Score (0–20)	Histological feature
0–4	Capillary congestion
0–4	Intra-alveolar hemorrhage
0–4	Perivascular hemorrhage
0–4	Interstitial neutrophil infiltration
0–4	Intra-alveolar neutrophil infiltration

Each feature was scored from 0 to 4 according to its severity, and the score for each feature was summed and averaged for each animal.

**Table 2:** Criteria for scoring lung fibrosis^45^.

Grade	Histological feature
0	Normal lung
1	Minimal fibrous thickening of alveolar or bronchiolar walls
2–3	Moderate thickening of walls without obvious damage to lung architecture
4–5	Increased fibrosis with definite damage to lung architecture and formation of fibrous bands or small fibrous mass
6–7	Severe distortion of the structure and large fibrous areas
8	Total fibrotic obliteration

### Immunohistochemical analysis

Paraffin-embedded tissue blocks were sectioned again into a positively charged slide. The sections were deparaffinized in an oven at 60 °C for 30 min, rehydrated via gradient ethanol, and treated with an unmasking solution for antigen retrieval. Endogenous peroxidase was inhibited with 3% H_2_O_2_ for 10 min at room temperature and away from light. The sections were incubated with a primary antibody at 4 °C overnight, and complete washing was performed by PBS, followed by a secondary antibody for 20 min and washing again. The positive immunostaining in the tissues was visualized by 3,3-diaminobenzidine tetrahydrochloride (DAB) and then stained with hematoxylin. The positive expression of the cell was brown^47^. The sections were observed by light microscope and photographed to estimate the expression of Smad3, fibronectin, vimentin, and α-SMA proteins. Semiquantitative scoring was used to evaluate the expression of the selected markers in the immuno-stained sections, and this was done by a pathologist blinded to the experimental groups. Details of the scoring method are explained in Table 3^48^.

**Table 3:** Immunohistochemistry scoring method^48^.

H-score (Histoscore)					
SI score	0	+ 1	+ 2	+ 3	+ 4
Positive cells (P)	< 10%	10–25%	25–50%	50–75%	> 75%

The H-score was calculated by a semiquantitative assessment of both the staining intensity (SI) and the percentage of positive cells (P). The range of possible scores was from 0 to 300. (H-score = SI * P).

### Cell culture and maintenance

MRC-5 (Human fetal lung fibroblast line) was purchased from the ATCC (American Type Culture Collection) organization (Virginia, United States). Cells were maintained in Dulbecco’s Modified Eagle Medium (EMEM) supplemented with 10% (v/v) fetal bovine serum (FBS) and antibiotics mixture (penicillin, streptomycin, amphotericin B) in standard culture conditions (5% CO_2_, 37 °C, 95% humidity). Cells were seeded into six-well plates, and their density was approximately 6 × 104 cells/mL, then incubated for 2–3 days to allow cells to grow and reach a confluency of about 70–80%.

### Determination of cell viability

The cytotoxicity of VCN in MRC-5 cells was assessed using an MTT assay. MRC-5 cells were seeded at a 5 × 10^3^ cells/mL density in 96-well plates. After growing for eight hours under normal growth conditions, cells were quiescent by incubating in a serum-free medium for 24 h and then treated with a normal medium containing serial concentrations of VCN (100, 50, 25, 12.5, 6.25 mcg/ml). Each concentration of the test agents was done in triplicates. All plates were covered and incubated in 5% CO_2_ in a humidified atmosphere at 37 °C for 24–48 h. As the negative control, MLB was added to the medium without cells. At the end of the drug-exposure period, the medium is removed from all wells containing cells. Plates are fed with 200 μL of fresh medium, and 50 μL of the MTT solution (5 mg/mL in PBS) is added to the wells. Plates were wrapped in aluminum foil and incubated for 4 h in a humidified atmosphere at 37 °C. The medium and MTT were then removed from the wells, and the purple MTT formazan crystals were dissolved by adding 200 μL of DMSO to all wells, followed by incubation at room temperature for 20 min. Absorbance was recorded at 570 nm using a Human^®^ microplate reader (USA)^49,50^.

The percentage of cytotoxicity for each of the assigned agents was calculated according to the formula:

$$Cytotoxicity \% =\frac{\left({OD}_{Control}- {OD}_{Sample}\right)}{{OD}_{Control}} \times 100\%$$

where OD control = optical density of the untreated cells, and OD sample = optical density of the treated cells.

### Transforming growth factor-β1 stimulated cells and treatments

After cells were grown and reached a confluency of 70–80%, cells were starved by changing the medium with EMEM containing 0.5% FBS and cultured again overnight^51^.

Cultured cells were grouped as follows for the specific experiment: the control group, which received the medium only; TGF-β1 group, which received the medium and 10 ng/ml of TGF-β1 only; TGF-β1 + PRD group, and TGF-β1 + VCN group. The last two groups were treated with the test agents, of which PRD served as the standard drug at a concentration of 100 µg/ml based on the results of the cytotoxicity MTT assay. Treated cells with the test agents received 10 ng/ml of TGF-β1 at the beginning and incubated for one hour, followed by adding the test agents supplemented in growth media. The cells were incubated for 48 h at 37 °C in a humidified CO_2_ 5% incubator. Each group was done in six replicates. At the end of the incubation period, plates were taken out and observed for any morphological changes with an inverted microscope^51^. Media was discarded, and cells were then collected and stored in TriZol^®^ reagent for subsequent gene expression analysis. The targeted genes in this experiment included (Vimentin, Fibronectin, α-SMA, and Smad2/3).

### RNA extraction and real-time quantitative PCR (RT-qPCR)

RNA was isolated from the cells and lung tissue samples according to the protocol of TRIzol^®^ Reagent (Thermo Scientific, USA). A Quantus Fluorometer (Promega, USA) was used to detect the concentration of extracted RNA. Total mRNA expression was measured using the SYBR Green kit and the one-step real-time qPCR analysis system (BioMolecular System. Australia) to determine the relative mRNA expression of (Nrf2, NLRP3, NF-κB, PPAR-γ) in mouse lung tissue and (Smad2/3, fibronectin, vimentin, and α-SMA) in MRC-5 cell line. All the values were normalized against the housekeeping gene (β-actin) with the delta-delta Cycle Threshold (DDCT) method. All the primers required for the analysis are listed in Table 4.

**Table 4:** List of forward and reverse primers required.

Gene	Forward primer	Reverse primer
(A) Primers required for *MRC-5 cell line* (*Homo Sapiens*) gene expression		
Fibronectin	CCACCCCCATAAGGCATAGG	GTAGGGGTCAAAGCACGAGTCATC
Vimentin	GCAAAGATTCCACTTTGCGT	GAAATTGCAGGAGGAGATGC
α-SMA	CAAAGCCGGCCTTACAGAG	AGCCCAGCCAAGCACTG
Smad2/3	GCCATCACCACTCAAAACTGT	GCCTGTTGTATCCCACTGATCTA
β-actin (RG)	ATCAAGATCATTGCTCCTCCTGAG	CTGCTTGCTGATCCACATCTG
(B) Primers required for *mouse lung tissue* (*Mus musculus*) gene expression		
Nrf2 gene	CAGCATAGAGCAGGACATGGAG	GAACAGCGGTAGTATCAGCCAG
PPAR-γ	GTACTGTCGGTTTCAGAAGTGCC	ATCTCCGCCAACAGCTTCTCCT
NF-κB	GCTGCCAAAGAAGGACACGACA	GGCAGGCTATTGCTCATCACAG
NLRP3	GGTGAGGCTGCAGTTGTCTA	CCCTTGGAGACACAGGACTC
β-actin (RG)	CATTGCTGACAGGATGCAGAAGG	TGCTGGAAGGTGGACAGTGAGG

*α-SMA* alpha-smooth muscle actin, *RG* reference gene/housekeeping gene, *Nrf2* nuclear factor erythroid 2-related factor 2, *PPAR-γ* peroxisome proliferator activated receptor-gamma, *NF-κB* nuclear factor-kappa B, *NLRP3* nucleotide-binding domain-like receptor protein 3 inflammasomes.

### Sample size calculation and animal randomization

For sample size computation, *program G Power* was utilized^52^ based on Cohen’s principles^53^. A table of random integers was used to construct the groupings at random. The animals were placed in labeled containers and given tail tags to minimize misunderstanding^54^.

### Statistical analysis

All data in the figures are presented as mean ± SD. Kruskal Wallis test and post hoc Dunn test were used to compare groups. The significance level was set at *P-values* ≤ 0.05. Data with *P-values* ≤ 0.01, 0.001, and 0.0001 were categorized as (*) significant, (**) high significant, and (***) very high significant. All data was analyzed using SPSS 29.0, while graphs and figures were created using GraphPad Prism 8.0.

## Results

### Vinpocetine alleviated fibrotic end points of bleomycin-induced pulmonary fibrosis

The effect of VCN on fibrogenesis was investigated in vivo using the mouse pulmonary fibrosis model, which was established by single-dose (5 mg/kg) bleomycin administered by oropharyngeal aspiration. Animals in the induced group that received only a single dose of BLM showed marked changes in their health status starting from day 4 of the induction with gradual worsening. Animals expressed a very low physical activity with a marked reduction in diet consumption, and animal fur became duller by the third week of the induction. Breathing patterns started to change by day 5 of the induction; animals showed rapid and shallow breathing, and a crackling sound during breathing was heard in a few of the animals by day 12, with worsened breathing by the end of the experiment. All animals in the induced group were in a hunched posture by the experiment’s end, and few showed cyanosis in the back and front hinges. These observations were markedly alleviated and improved in VCN-treated animals.

**Figure 2:**
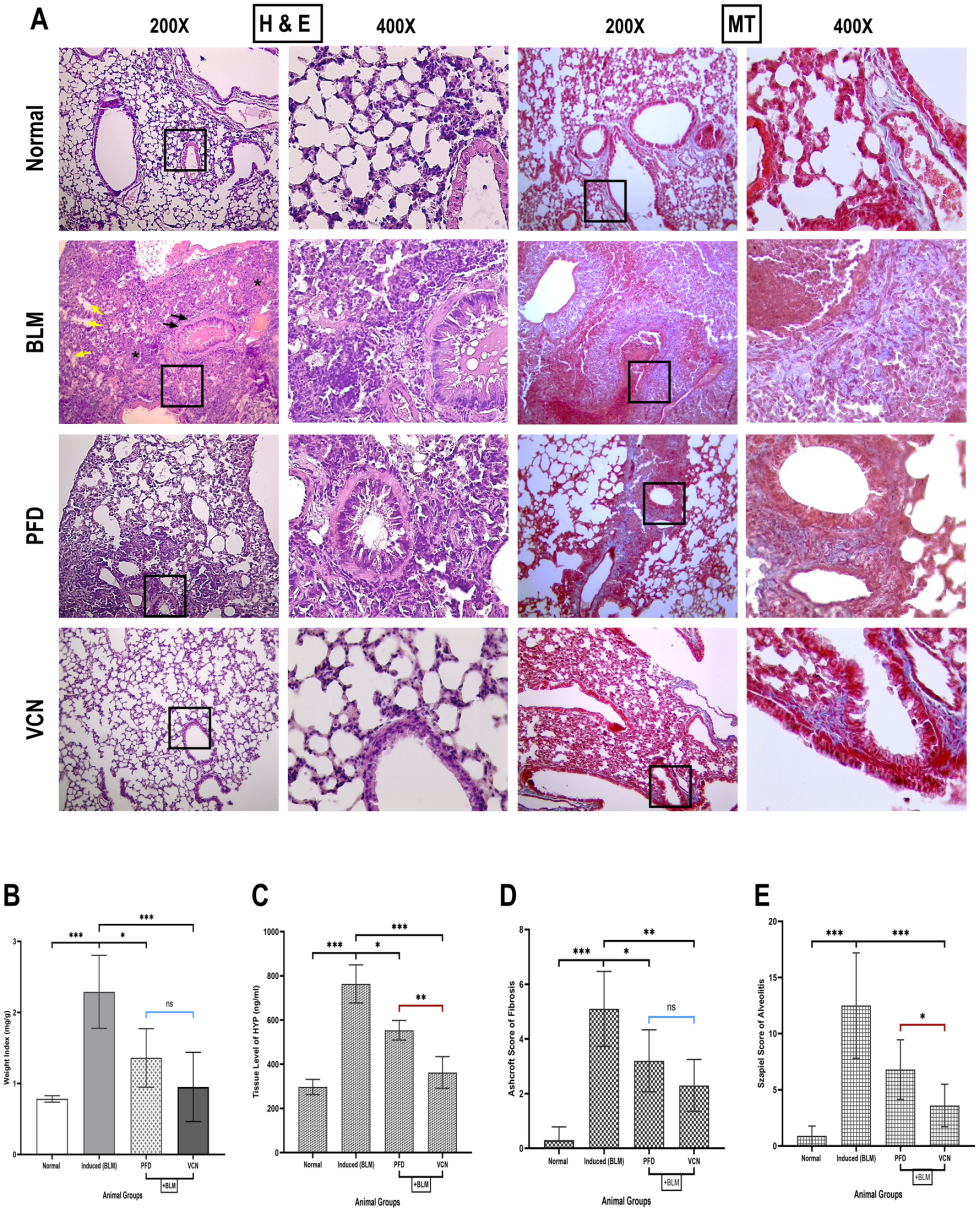
Vinpocetine reduced major fibrotic endpoints of BLM-induced pulmonary fibrosis. (**A**) represents lung tissue sections stained with (H & E) and (MT) (200X, and 400X), (**B**) represents changes in lung weight index, (**C**) represents changes in lung tissue content of hydroxyproline, (**D**) represents the Ashcroft score of fibrosis, and (**E**) represents Zsapiel score of alveolitis [Results are expressed as mean ± SD, (n = 10). Significance was set at (P ≤ 0.05). *(P ≤ 0.01), **(P ≤ 0.001), and ***(P ≤ 0.0001)] (*BLM* bleomycin, *PFD* pirfenidone, *VCN* vinpocetine, *HYP* hydroxyproline, *MT* Masson Trichome, *H & E* Hematoxylin and Eosin) [White arrow = capillary congestion and hemorrhage, Black arrow = increased thickness of the bronchiolar wall, yellow arrow = loss of alveolar spaces, star = inflammatory infiltrate].

Regarding lung weight index, at the end of the experiment (day 22), animals were sacrificed, and lungs were harvested and weighed; the weight index was calculated based on the equation provided previously. The results showed that there is a statistically significant elevation in the lung weight index in the induced group compared to the normal (P = 0.0001); on the other hand, the lung weight index of animals treated with PFD (standard treatment) and VCN was significantly reduced (P = 0.02 and P = 0.0001) with better outcomes for the latter [Fig. 2B]. Hydroxyproline (HYP) measurement was an important indicator of tissue remodeling and excessive collagen deposition in different tissues. HYP is an amino acid present in collagen after hydroxylation of the proline moiety and represents about 13%—14% of the total amino acid content in collagen^55^. Results of lung tissue lysate analysis revealed a statistically significant elevation of HYP in the induced (BLM) group compared to the normal (P = 0.0001), which confirms the successful induction of the pulmonary fibrosis model. Meanwhile, VCN significantly improved the lung content of HYP (P = 0.0001) with much lower values than that of PFD (P = 0.008) [Fig. 2C]. The changes in lung HYP level were further supported by the changes observed in the histopathological features.

Lung tissue sections stained with H & E revealed marked damage to the lung architecture in lung tissue of animals in the induced (BLM) group with severe collapse and loss of the alveolar spaces, capillary congestion with signs of hemorrhage, and increased interstitial infiltration of inflammatory cells compared to the normal group of animals which maintained a normal lung architecture with minimal to no evident of alveolar or bronchial wall thickening [Fig. 2A] and was further supported by Szpiel scoring for alveolitis which showed a statistical elevation in the score (P ≤ 0.0001) of the BLM-only treated animals (12.50 ± 4.70) compared to the normal animals [Fig. 2E]. PFD (standard treatment and VCN were able to alleviate the damaging effects produced by BLM with varying degrees, where lung sections of VCN-treated animals showed better histological observations compared to PFD, which was observed in the alveolitis score as well. On the other hand, MT-stained lung tissue sections to assess collagen deposition and the extent of fibrosis revealed that the BLM-only treated group expressed a marked increase in collagen deposition with the distribution of collagen fibers around the bronchi in the interstitium. Some alveolar spaces were filled with collagen fibers. This increase in collagen deposition was semi-quantified by the Ashcroft score of lung fibrosis. It was found to be moderately elevated (5.10 ± 1.37) in comparison to the normal group, which showed minimal collagen deposition (as indicated by the faint blue color) and a very low score (0.30 ± 0.48), which confirms a significant deviation and damage to the structural integrity of the lung (P = 0.0001) as shown in [Fig. 2A,D)]. By contrast, groups that received PFD (standard treatment) and VCN managed to improve lung architecture and reduce collagen deposition, which correlates with a significant improvement in the Ashcroft score (3.20 ± 1.14, P = 0.04) and (2.30 ± 0.95, P = 0.001) respectively with slightly better outcomes for VCN.

**Figure 3:**
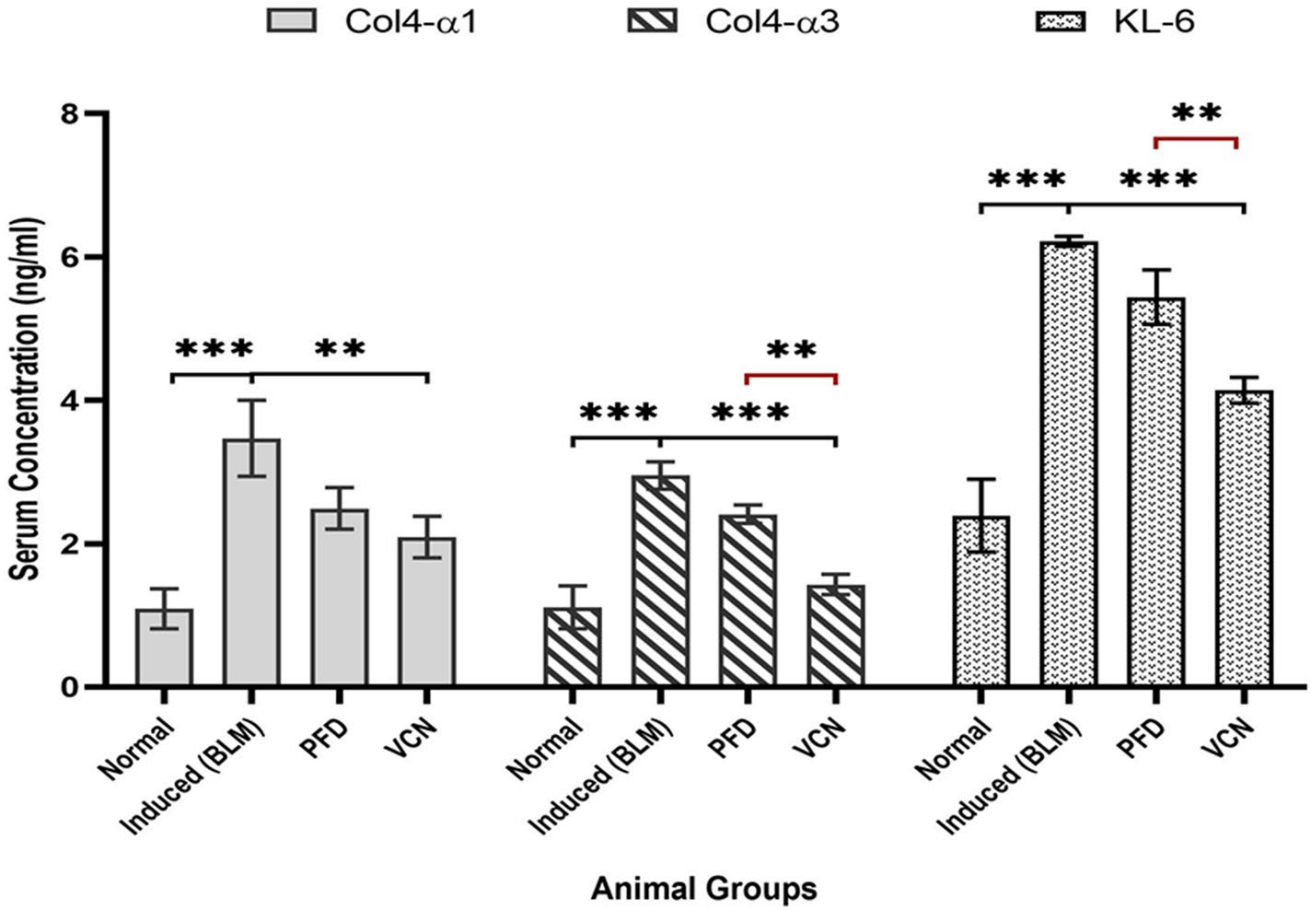
Changes in serum level of fibrotic markers including (ColIVα1, ColIVα3, and KL-6) among animal groups. [Results are expressed as mean ± SD, (n = 10), and significance was set at (P ≤ 0.05). *(P ≤ 0.01), **(P ≤ 0.001), and ***(P ≤ 0.0001), *ns* no significance.], (*BLM* bleomycin, *PFD* pirfenidone, VCN vinpocetine, *Col4α1* collagen type IV alpha1, *Col4α3* collagen type IV alpha3, *KL-6* Krebs von den lungen-6).

Other fibrotic endpoints were measured in serum samples, including collagen type IV-α1 (ColIVα1), collagen type IV-α3 (ColIVα3), and Krebs von den lungen-6 (KL-6). The results of serum analysis revealed that all three markers were significantly elevated in the induced (BLM) group compared to the normal group (P = 0.0001), as demonstrated in [Fig. 3]. VCN managed to alleviate the levels of all three markers produced by the BLM challenge (P ≤ 0.001) with statistically better effects than PFD regarding ColIVα3 and KL-6 [Fig. 3].

### Vinpocetine reduced ECM deposition in immuno-stained lung tissue section

The immunohistochemistry technique was used to evaluate the expression of certain specific fibrotic markers involved in the activation and differentiation of fibroblasts and deposition of ECM, including fibronectin, vimentin, alpha-smooth muscle actin (α-SMA), and Smad3 (an intracellular signaling protein in the activation of fibroblasts). Changes in protein expression (increased or decreased) were observed as the brown pigment in the section, in which the level of expression was quantified using a scoring system (H-score) that depends on the staining intensity and percentage of positive cells as explained in [Table (1)].

**Figure 4:**
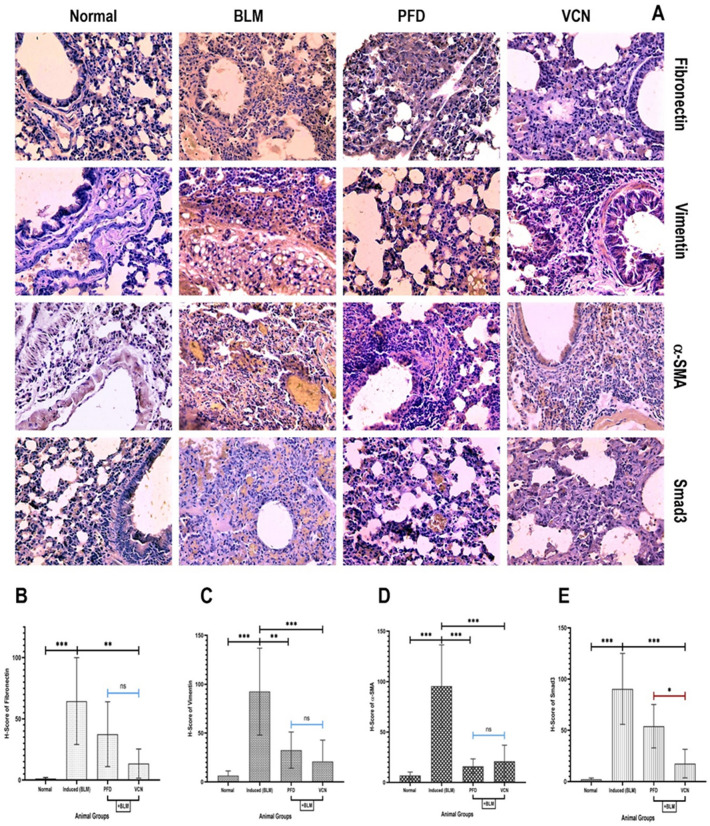
Changes in fibrotic-related markers in immunohistochemistry-stained lung tissue section. (**A**) immunohistochemical stained images of (fibronectin, vimentin, α-SMA, and Smad3) where protein expression is indicated in areas of brown pigment (400X), H-scores of protein expression of (**B**) fibronectin, (**C**) vimentin, (**D**) α-SMA, and (**E**) Smad3. [Results are expressed as mean ± SD, (n = 10), and significance was set at (P ≤ 0.05). *(P ≤ 0.01), **(P ≤ 0.001), and ***(P ≤ 0.0001), *ns* no significance.], (*BLM* bleomycin, *PFD* pirfenidone, *VCN* vinpocetine, *α-SMA* alpha-smooth muscle actin).

The results of the analysis revealed a significant increase in the expression/deposition of the mentioned fibrogenic elements in the BLM-only treated animals (induced group) (P < 0.0001) compared to the normal group, with a marked increase in the brown-stained areas of the alveolar septa where fibronectin and vimentin are likely to deposit and α-SMA and Smad3 are likely distributed in areas of fibroblasts and myofibroblasts as seen in [Fig. 4A, BLM]. The VCN-treated group showed a remarkable reduction in all the fibrogenic markers in comparison to the induced group and with statistical significance [fibronectin, P = 0.003), (vimentin, P = 0.0002), (α-SMA, P = 0.0007), (Smad3, P = 0.0004)]. In contrast, in the PFD-treated group, there was only a significant reduction in vimentin (P = 0.007) and α-SMA (P = 0.0004) compared to the induced (BLM) group, as shown in [Fig. 4C,D].

### Vinpocetine reduced lung tissue levels of pro-fibrotic and inflammatory cytokines

**Figure 5:**
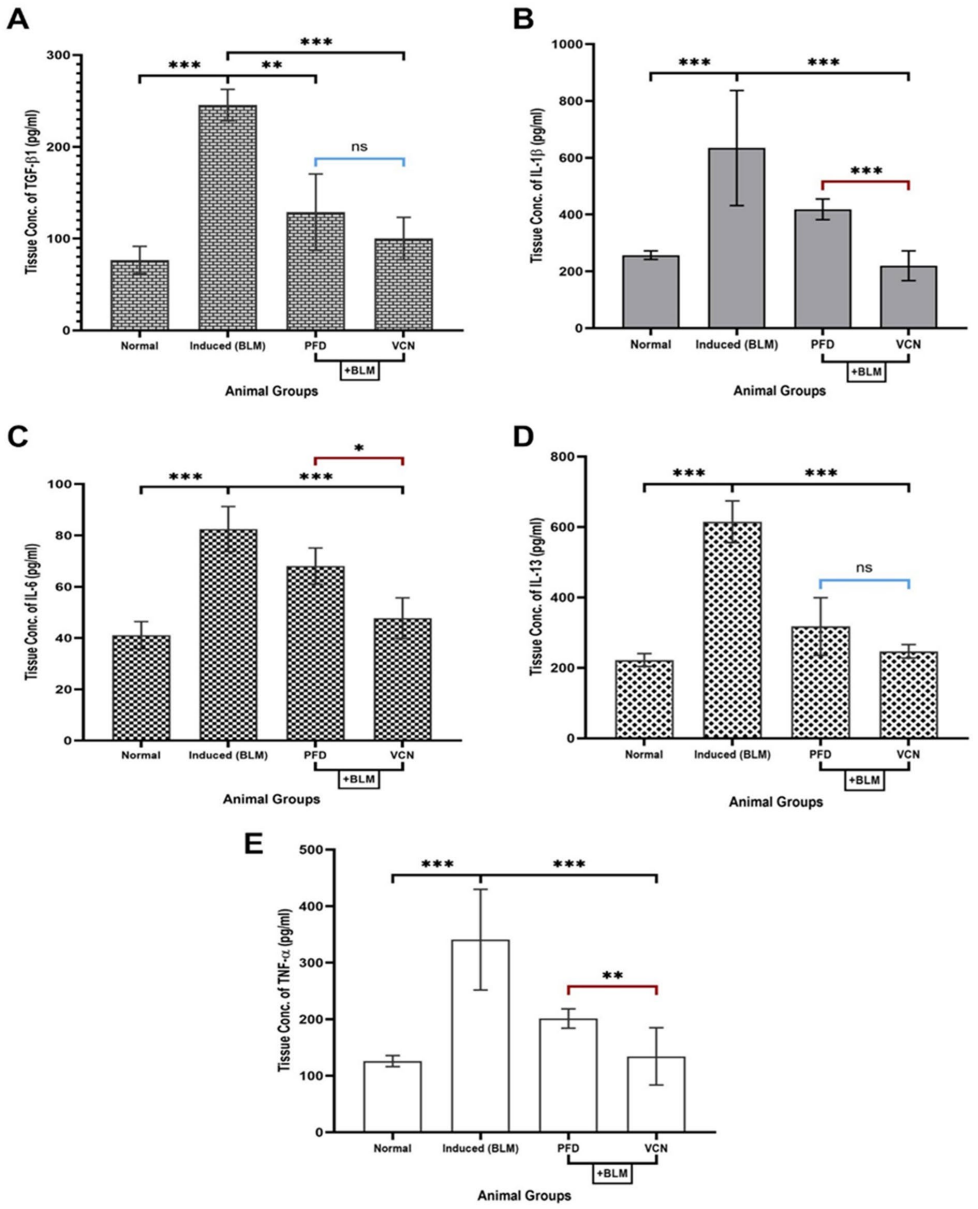
Changes in pro-fibrotic and inflammatory cytokines involved in the pathogenesis of pulmonary fibrosis among animal groups. Lung tissue level of (**A**) TGF-β1, (**B**) IL-1β, (**C**) IL-6, (**D**) IL-13, and (**E**) TNF-α. [Results are expressed as mean ± SD, (n = 10), and significance was set at (P ≤ 0.05). *(P ≤ 0.01), **(P ≤ 0.001), and ***(P ≤ 0.0001), *ns* no significance]. (*TGF-β1* transforming growth factor-beta1, *IL* interleukin, *TNF-α* tumor necrosis factor-alpha, *BLM* bleomycin, *PFD* pirfenidone, *VCN* vinpocetine).

The most important cytokines that were measured in the study and found to be involved in the development of PF include (TGF-β1, IL-1β, IL-6, IL-13, and TNF-α). These mediators were measured in lung tissue homogenate samples using the ELISA technique. The results of the study revealed that there was a statistically significant elevation (P = 0.0001) in the levels of all the mentioned cytokines in the induced group (treated only with BLM/ET) compared to the normal group, which suggests a successful induction of the pulmonary fibrosis model [Fig. 5]. VCN managed to significantly produce better outcomes for all the mentioned cytokines (P ≤ 0.05); by contrast, the animal group treated with the standard anti-fibrotic agent (PFD) showed a significant reduction only for TGF-β1 compared to the induced group (P = 0.002) as shown in [Fig. 5A]. VCN expressed far better results in comparison to PFD by showing a statistically significant reduction in the levels of IL-β1 (P = 0.0001), IL-6 (0.02), and TNF-α (0.004).

### Vinpocetine alleviated lung tissue oxidative stress produced by the BLM challenge

Measurement of biomarkers related to oxidative stress was done in lung tissue lysate using the ELISA technique. Selected markers that reflect oxidative stress and antioxidant activity were malondialdehyde (MDA), glutathione (GSH), superoxide dismutase (SOD), and glutathione peroxidase (GPx).

**Figure 6:**
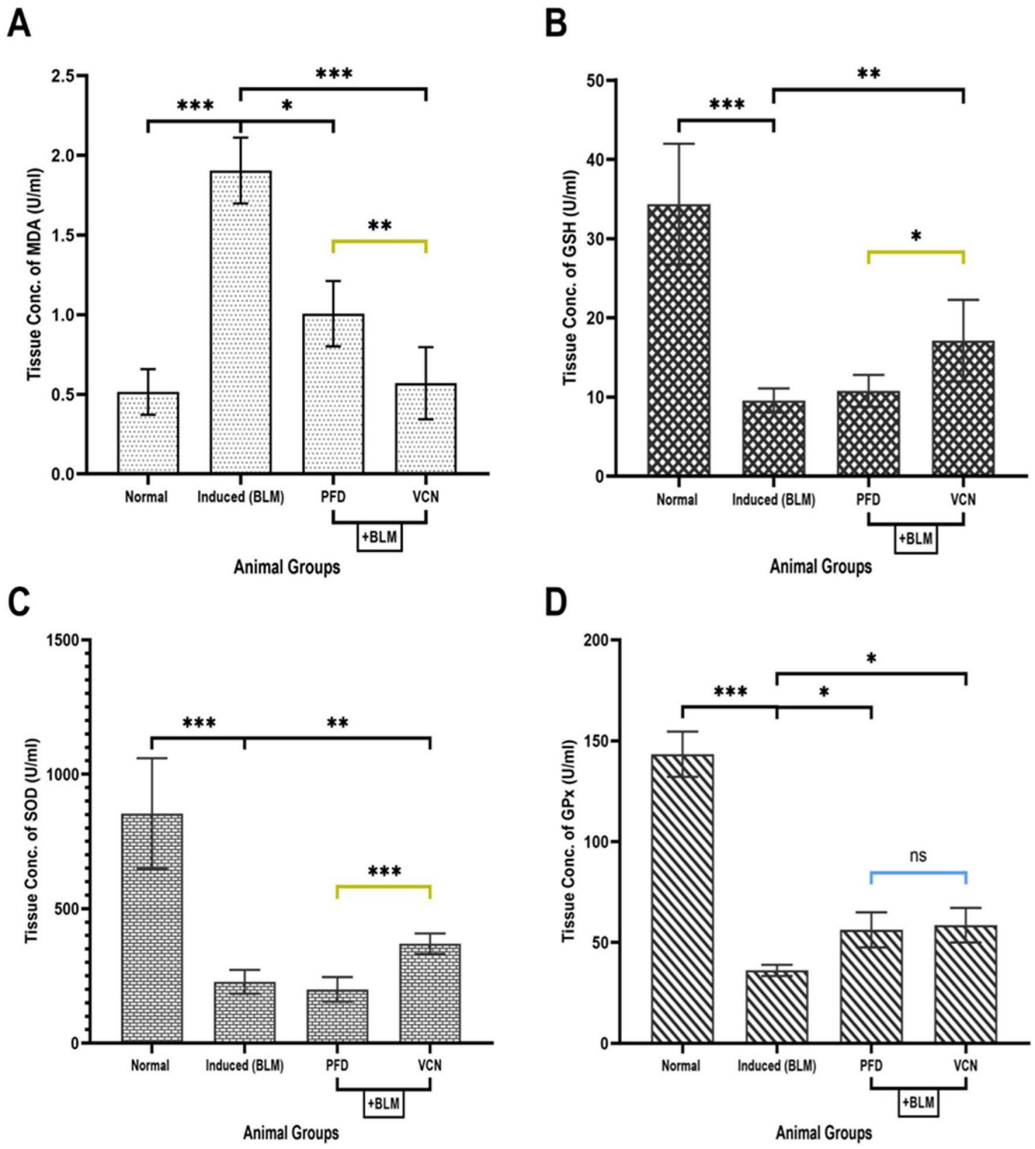
Changes in tissue oxidative stress parameters among animal groups. The Figure represents lung tissue levels of (**A**) MDA, (**B**) GSH, (**C**) SOD, and (**D**) GPx. [Results are expressed as mean ± SD, (n = 10), and significance was set at (P ≤ 0.05). *(P ≤ 0.01), **(P ≤ 0.001), and ***(P ≤ 0.0001), *ns* no significance], (*BLM* bleomycin, *PFD* pirfenidone, VCN vinpocetine, *MDA* malondialdehyde, *GSH* glutathione, *SOD* superoxide dismutase, *GPx* glutathione peroxidase).

The result of the analysis demonstrated a statistically marked elevation in the oxidative biomarker (MDA) and a significant reduction in the antioxidant biomarkers (GSH, SOD, and GPx) in the induced (BLM) group (P = 0.0001) compared to the normal animals [Fig. 6] which confirms a successful induction of the model by disrupting the balance of the antioxidant system due to the general mechanism of the inducing agents (BLM). Regarding the oxidative biomarker (MDA), animal groups treated with PFD (standard treatment) and VCN demonstrated a statistically significant reduction in the level of MDA compared to the induced group with p-value (P = 0.05 and P = 0.0001), respectively, and VCN-treated animals showed a better reduction in MDA compared to standard treatment (P = 0.005) as demonstrated in [Fig. 6A].

Regarding the antioxidant biomarkers (GSH, SOD, and GPx), animals that received PFD (standard treatment) showed a significant elevation only in the level of GPx compared to the induced (BLM) group (P = 0.04) [Fig. 6D]. In contrast, animals treated with VCN demonstrated better results with statistically significant elevation in the levels of all antioxidant biomarkers (P ≤ 0.01). Compared to standard treatment (PFD), animals treated with VCN showed statistically better results in the levels of (GSH, P = 0.01) and (SOD, P = 0.0001) [Fig. 6B and C].

### Vinpocetine alleviated lung tissue mRNA expression of inflammatory and oxidative stress genes

**Figure 7:**
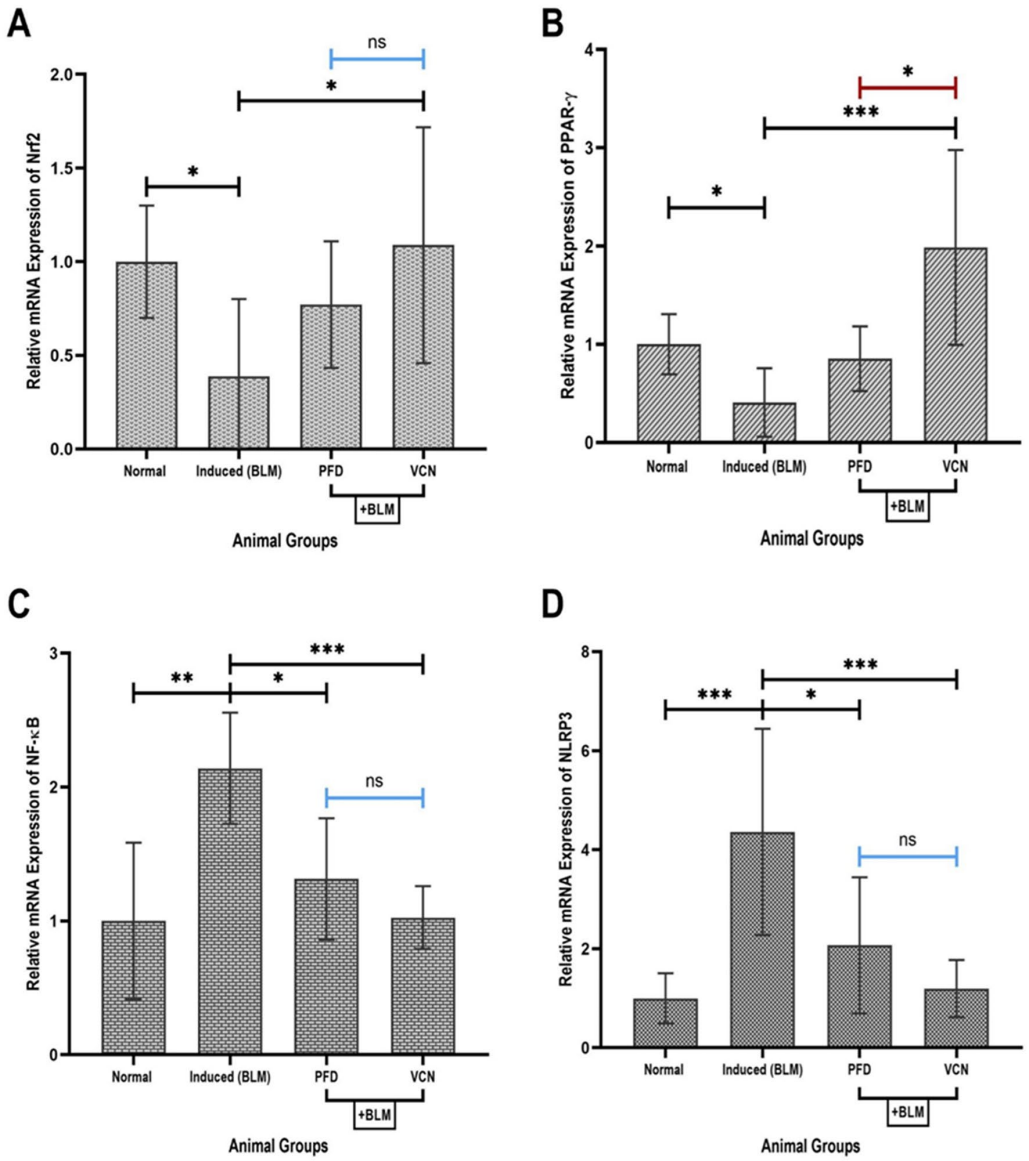
Changes in relative mRNA expression in lung tissue of different animal groups. The Figure represents the relative expression of mRNA of (**A**) Nrf2, (**B**) PPAR-γ, (**C**) NF-κB, and (**D**) NLRP3. [Results are expressed as mean ± SD, (n = 7), and significance was set at (P ≤ 0.05). *Represents (P ≤ 0.01), **Represents (P ≤ 0.001), and ***Represents (P ≤ 0.0001), *ns* no significance, results were normalized to β-actin as the housekeeping gene], (*BLM* bleomycin, *PFD* pirfenidone, *VCN* vinpocetine, *Nrf2* nuclear erythroid factor-related factor2, *PPAR-γ* peroxisome proliferator-activated receptor-gamma, *NF-κB* nuclear factor-kappa B, *NLRP3* nucleotide-binding domain-like receptor protein three inflammasomes).

Gene expression was analyzed using the RT-qPCR technique. Mouse lung tissue was excised, cleansed, and cut into two parts, about 50mg of the right lung’s distal region, for gene expression assessment. Seven samples in each group were included in the final assessment due to unwanted contamination and RNA extraction errors. Among the most important genes selected for the study is Nrf2, which regulates the capacity of the antioxidant system. In contrast, PPAR-γ, NF-κB, and NLRP3 regulate the inflammatory immune response. Evaluation of mRNA level was as the relative expression of a specific gene, where the result of each specific gene was normalized to the housekeeping gene (β-actin). Analysis of the relative gene expression revealed that animals that received only BLM challenge by oropharyngeal administration (induced group) showed a significant reduction in the expression of Nrf2 (P = 0.01) and PPAR-γ mRNA (P = 0.04). At the same time, there was a marked elevation in the expression of mRNA of NF-κB (P = 0.0017) and NLRP3 (P = 0.0003), as shown in [Fig. 7]. Compared to the induced group, VCN significantly improved the expression of all the mentioned genes (P ≤ 0.05). In contrast, PFD was able to significantly reduce the expression of NF-κB (P = 0.01) and NLRP3 (P = 0.02) [Fig. 7C,D].

### Vinpocetine inhibited TGF-β1 induced fibroblasts to myofibroblasts differentiation through TGF-β1/Smad pathway

**Figure 8:**
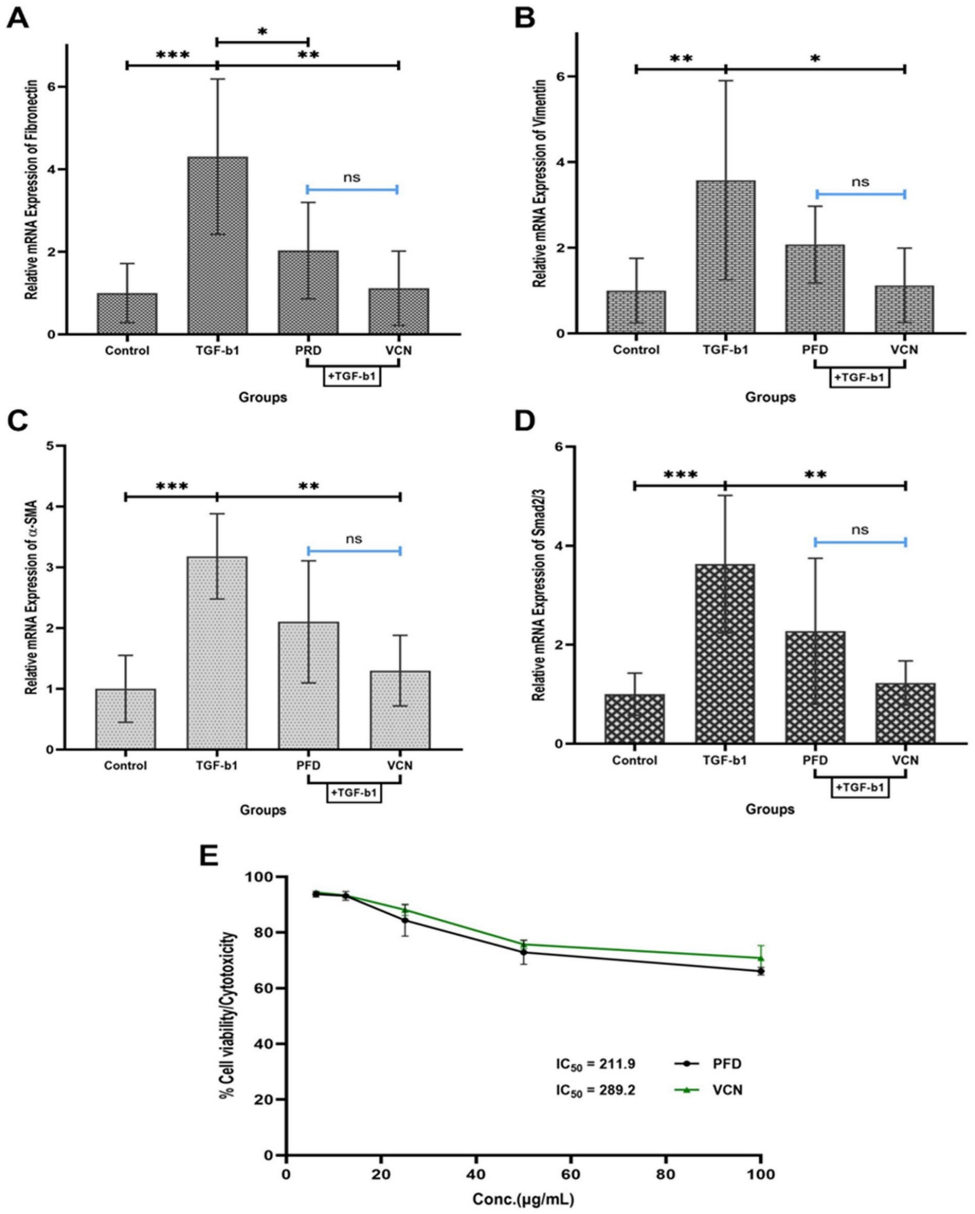
Vinpocetine inhibited TGF-β1 induced fibroblasts to myofibroblasts differentiation using the MCR-5 cell line. Changes in relative mRNA expression of (**A**) fibronectin, (**B**) vimentin, (**C**) α-SMA, (**D**) Samd2/3, and (E) represent MRC-5 viability after treatment with serial concentrations of VCN. [Results are expressed as mean ± SD, (n = 6, RT-qPCR), (n = 3, MTT assay), and significance was set at (P ≤ 0.05). *Represents (P ≤ 0.01), **Represents (P ≤ 0.001), and ***Represents (P ≤ 0.0001), *ns* no significance, results of RT-qPCR were normalized to β-actin as the housekeeping gene], (*BLM* bleomycin, *PFD* pirfenidone, *VCN* vinpocetine, *Nrf2* nuclear erythroid factor-related factor2, *PPAR-γ* peroxisome proliferator-activated receptor-gamma, *NF-κB* nuclear factor-kappa B, *NLRP3* nucleotide-binding domain-like receptor protein three inflammasomes).

Given the anti-inflammatory and anti-fibrotic effect of VCN on pulmonary fibrosis, the study aimed to define the molecular mechanism underlying the role of VCN in BLM-induced fibrosis. Cell viability was assessed at the beginning using the MTT assay. Five serial concentrations for PFD and VCN were used in the assay (100, 50, 25, 12.5, and 6.25) µg/ml. Results revealed that PFD and VCN exhibited non-toxic effects on the MRC-5 cell line with IC50 (211.9 µg/ml) and (289.2 µg/ml), respectively, as shown in [Fig. 8E)].

To elucidate the probable mechanism of action of the anti-fibrotic activity of VCN observed during the in vivo experiment, human fetal lung fibroblasts (MRC-5) were cultured into four groups (control, induced, induced + PFD, and induced + VCN) of 6 replicates to study the expression of certain genes involved in the fibrogenesis including (fibronectin, vimentin, α-SMA, and Smad2/3). Groups of cultured cells were pretreated with TGF-β1 (10ng/ml) for one hour except for the control group, followed by treatment with PFD (standard) and VCN at a concentration (100 µg/ml) based on the results obtained from the viability/cytotoxicity analysis by MTT. Forty-eight hours after incubation with the treatment, cultured cells were collected with TRIzol® reagent for RNA extraction and gene expression analysis. Results of MRC-5 cells gene expression showed that cells treated only with TGF-β1exhibited significant elevation in the expression of all fibrotic genes (P ≤ 0.0001) compared to the control group. PFD and VCN reduced gene expression of fibrotic markers in treated cells with varying levels compared to TGF-β1-only treated cells. Although PFD managed to reduce the expression of all the fibrotic markers, a statistically significant reduction was only shown for fibronectin (P = 0.02) [Fig. 8A]. VCN expressed better results than PFD in reducing fibrotic gene expression with high statistical significance compared to the induced cell (P ≤ 0.001).

## Discussion

The animal models of pulmonary fibrosis are still an irreplaceable instrument for the comprehensive study of etiological factors, molecular mechanisms, possible markers, and potential therapeutic targets of lung tissue remodeling and fibrogenesis. No animal model of pulmonary fibrosis can recapitulate all the pathological aspects of human PF. Murine models of PF are the most commonly used to study any agent with a potential anti-fibrotic effect^12^. Bleomycin is an antitumor antibiotic that damages the cells through single- or double-strand DNA breaks, leading to cell cycle arrest^56^. BLM can replicate about 70–80% of the human pathological PF by causing direct cell damage, free radical production, and the development of oxidative stress, followed by necrosis or apoptosis of epithelial and endothelial cells, lung inflammation, and, as a result, pulmonary fibrosis development^12,57^.

BLM can be administered by different routes for the induction of pulmonary fibrosis; however, intratracheal (IT) is the most common route of administration. IT administration is believed to recapitulate better the human phenotype limited to the lungs^58^. The endotracheal/oropharyngeal method of delivery was used in this study. An alternative to the IT method is the non-invasive endotracheal/orotracheal/oropharyngeal aspiration (ET/OT/OP), which delivers BLM through the trachea with minimal associated side effects and mortality^59^.

VCN was chosen for this study because of its promising biological profile of anti-inflammatory and antioxidant activities through modulating the activity of the expression factors NF-κB and Nrf2. The dose selected for VCN was (20 mg/kg) based on previous study^35^. According to acute toxicity data, where the oral LD50 (lethal dose for 50% of exposed animals) value for VCN is approximately 500 mg/kg in rats and mice, the intravenous LD50 is approximately 50 mg/kg in rats and mice. The intraperitoneal LD50 ranges from 134 to 240 mg/kg in rats and mice^60^.

Many cytokines are involved in the development of PF. TGF-β1 is the central regulator in the progression of PF. TGF-β can stimulate trans-differentiation in fibroblasts, converted into myofibroblasts with the upregulation of alpha-smooth muscle actin (α-SMA), eventually contributing to fibrotic foci in lung tissue^61^. On the other hand, the type II alveolar epithelial cells (AEC II) in lung tissue can also be stimulated by TGF-β to trans-differentiate into fibroblasts^33^, with a loss of the epithelial phenotype and mesenchymal feature acquisition, which is known as epithelial-to-mesenchymal transition (EMT)^39^. When the TGF‑β1 signal activated TGF‑β type I receptor kinase, R‑Smads (Smad2 and Smad3) were phosphorylated, where Smad3 is more sensitive to TGF‑β1 than Smad2^62^. Activated Smad2 and Smad3 form a complex, which combines with the Co‑Smad (Smad4) and transfers into the nucleus to regulate the expression of target genes. The contribution of the TGF‑β1/Smad signaling pathway to PF mainly depends on the following three processes: Myofibroblast differentiation, EMT/EndMT, and fibrogenesis^63^. These explanations support the findings of elevated tissue levels of TGF-β1 and expression of Smad3 in the BLM group, while they were significantly reduced in the animals treated with VCN.

To confirm the establishment and improvement of lung fibrosis after the challenge with BLM and treatment with VCN, certain markers were selected for this purpose which includes tissue collagen deposition determined by histological scoring of fibrosis and measurement of tissue hydroxyproline (an indicator for newly formed collagen), measurement of serum level of collagen type IV and KL-6, as well as determination of presence of fibronectin, vimentin, and α-SMA by immunostaining which are markers for ECM synthesis and indicators for myofibroblasts formation (especially α-SMA). BLM-only treated animals showed a marked elevation in hydroxyproline (HYP) lung tissue content, which supports the marked increase in the lung weight index. In contrast, HYP level was alleviated in the animal group that received VCN; this was further supported by the histopathological investigation of lung tissue collagen deposition using MT staining and Ashcroft fibrosis score. Levels of KL-6 (a marker for AEC injury), ColIVα1, and ColIVα3 (markers for BM destruction) were significantly elevated in the serum of animals in the induced group, signifying that BLM caused AEC damage that resulted in the release of KL-6^23^ and recruitment of inflammatory cells (e.g., monocytes) leading to the release of MMPs which results in further degradation of the surrounding BM and the release of collagen type IV fragments into the blood circulation^58,64^.

On the other hand, treated animals with test agents (VCN) showed a statistically significant reduction in these biomarkers. Fibronectin, vimentin, and alpha-smooth muscle actin (α-SMA) are three important ECM proteins that play key roles in the pathogenesis of pulmonary fibrosis. They are also considered markers for mesenchymal transitioning and myofibroblast formation. Fibronectin and vimentin are important for fibroblast migration, activation, enhanced inflammation through NLRP3, and epithelial-mesenchymal transition through activating the TGF-β1/Smad pathway^65^. BLM increased the level of these ECM proteins in the immune-stained lung sections, whereas VCN managed to dampen their expression. These results support the data obtained regarding the tissue level of TGF-β1 [Fig. 4A] and Smad3 [Fig. 3E] being the main players in the development and progression of pulmonary fibrosis. Additionally, this proves that VCN managed to reduce the production of ECM by inhibiting the formation of myofibroblasts and epithelial-mesenchymal transitioning, probably via interfering with the TGF-β1/Smad3 signaling pathway.

The reduction in the tissue level of the pro-fibrotic TGF-β1 in the VCN-treated group was consistent with a previous study published by Balaha et al.^66^. Also, the results of IL-6 and TNF-α in the induced (BLM) group and the VCN-treated group were supported by the findings of Balaha et al. in studying the protective effect of different doses of VCN in experimental pulmonary fibrosis in rats—the same observations regarding the oxidative markers (MDA, GSH, and SOD). In the context of IL-13, the results of the VCN-treated animals were supported by a previously published work investigating the anti-inflammatory effect of 10mg/kg of vinpocetine in a model of allergic asthma in mice^30^. In the context of altering the lung tissue expression of NF-κB and the inflammasome NLRP3, the results of the present work regarding the induced (BLM) group and the VCN-treated group were supported by a recently published study on the ameliorating effect of vincamine in pulmonary fibrosis in mice^67^.

Cytokines are important biomolecules in inflammation and immune response and are proven to be involved in the formation and development of pulmonary fibrosis. Among the most important cytokines involved in tissue injury/repair process and were the center of focus in this study (IL-1β, IL-6, IL-13, and TNF-α). During lung injury development, the destruction of AEC II leads to a significant decrease in surfactant production, and as a result, alveoli collapse, and lung proteins permeate into the alveoli space^68^. IL-1β is one of the secreted mediators from alveolar macrophages in response to activation of the cytosolic inflammasome (NLRP3) after priming with certain stimuli. Activated inflammasomes will cleave pro-IL-1β into its active form through caspase-1^69^. IL-1β is a potent pro-inflammatory cytokine that can initiate and amplify lung inflammation and has been associated with acute lung injury by enhancing the recruitment of neutrophils, monocytes, and lymphocytes, precipitating fibrosis through activation of resident pulmonary fibroblasts to synthesize collagen and fibrin^69^.

Interleukin-6 (IL-6) is another important cytokine. In response to the BLM challenge, injury to endothelial cells and AECs takes place, causing the release of inflammatory mediators, one of which is IL-6. As a result of its release, the formation of IL-6/sIL-6Ra complex stimulates IL-6 trans-signaling in lung fibroblasts and other cells to promote proliferation and ECM formation^70^. IL-6 and Th2 cell cytokines (IL-4, IL-5, and IL-13) will activate M2 macrophages and change their phenotype into hyper-profibrotic macrophages that induce ECM synthesis and further aggravate pulmonary fibrosis^70,71^. IL-6 stimulates and activates resident fibroblasts through the ERK/STAT3 signaling pathway and changes their phenotype into apoptosis-resistant by increasing the expression of the anti-apoptotic molecule Bcl-2^72^. TNF-α can influence the release and activity of IL-13 from macrophages and the recruitment of other inflammatory cells into the injury site. Still, it influences the release of TGF-β1, the activation and proliferation of fibroblasts, and their differentiation into myofibroblasts, mostly mediated by the RANKL/NF-κB pathway. Secreted IL-13 from activated Th2 cells can influence the release of TGF-β1 from activated macrophages through the JNK signaling pathway, regulate fibroblast proliferation and differentiation through the JNK and AKT signaling pathway as well and promote the expression of α-SMA and collagen I that leads to the development of fibrosis^71^; this explains the findings of this study, where tissue levels of the selected pro-inflammatory mediators were all elevated in BLM-only-treated animals, proving the fact that BLM caused significant alveolar epithelial damage that initiates a phase of acute inflammatory response in which innate immunity is the main player, especially during the first week of the induction followed by the activation of the adaptive immunity and the release of important cytokines to activate and propagate fibrosis of the pulmonary architecture^12^. VCN exhibited great improvement in the levels of pro-inflammatory cytokines compared to the induced group. This significant improvement in the inflammatory activity could be because VCN is a phosphodiesterase 1 (PDE1) inhibitor. PDE1B activation promotes the differentiation of monocytes to macrophages; thus, the anti-inflammatory activity of VCN is likely attributed to its inhibitory effect of PDE1 with higher affinity for PDE1A and PDE1B isoforms^73^. A recent study that investigated the effect of VCN in suppressing the progression of non-alcoholic steatohepatitis (NASH) in mice mentioned that the anti-inflammatory of VCN could be mediated by inhibition of NLRP3 inflammasome activation and hence the NF-κB signaling pathway which results in a reduction in the production of pro-inflammatory cytokines^74^. Zhang and colleagues mentioned that VCN can inhibit IκB kinase (IKK), which plays a critical role in cellular inflammatory response and increases expression of NF-κB^24^.

Elevated tissue inflammatory cytokines to BLM challenge and the anti-inflammatory activity of VCN were further explained and greatly supported by the results of lung tissue gene expression of (NLRP3, NF-κB, and PPAR-γ). PPAR-γ is a nuclear receptor that regulates different metabolic pathways and inflammatory responses, where activation of this receptor can interfere with TGF-β1 signaling via the Smad pathway and suppress the production of inflammatory mediators by downregulating NF-κB. Thus, studies have suggested that peroxisome proliferator-activated receptor gamma (PPAR-γ) may play a key role in modulating the inflammatory and fibrotic responses in the lung^19,75^. Again, the effect of VCN on the elevated expression of PPAR-γ could be attributed to being a direct inhibitor of PDE1. Reports have shown that PPAR-γ and PDE1 have a complex relationship that affects various physiological and pathological processes. Inhibition of PDE1 can enhance the expression and activity of PPAR-γ, which improves glucose and lipid metabolism and anti-inflammatory activity supported by previous studies^76,77^. NLRP3 and NF-κB complement one another, where NLRP3 is a cytosolic protein complex that forms an inflammasome in response to various stimuli, such as pathogens, toxins, or cellular stress. As noted earlier, NLRP3 inflammasome activation can trigger the cleavage and secretion of IL-1beta and IL-18, potent pro-inflammatory and pro-fibrotic cytokines. Additionally, it is an important pathway in activating the nuclear transcriptional factor NF-κB^78^. NF-κB activation can induce the expression of pro-inflammatory cytokines, chemokines, adhesion molecules, and matrix metalloproteinases, which contribute to the recruitment of inflammatory cells, tissue damage, and remodeling in PF^18^.

Results of the lung tissue oxidative stress analysis showed that bleomycin (BLM) caused a significant elevation in the oxidative stress biomarker (MDA), an end product of lipid peroxidation, and a reduction in the counter-antioxidant defense markers (GSH, SOD, and GPx) which could be related to one of the molecular mechanisms of injury of BLM by generating a high level of ROS and disturbing the balance of oxidant/antioxidant system. Several studies have reported that oxidative stress was associated with the development of alveolar injury, inflammation, and fibrosis^79^. MDA can influence the inflammatory response of the innate immune system through several signaling pathways, including protein kinase-C, p38-MAPK, ERK1/2, and NF-kB^80^. Oxidative stress might promote a fibrotic microenvironment, where fibroblasts were resistant to the damaging effects of ROS in PF, but epithelial cells were relatively more sensitive to oxidative stress. Lung myofibroblasts are known to secrete hydrogen peroxide, leading to mediated fibrogenic effects, and inducing epithelial apoptosis^79^.

Moreover, recruited neutrophils during the induction’s inflammatory phase (first week) will further participate in tissue injury and fibrosis by producing matrix-degrading enzymes such as neutrophil elastase and releasing excessive ROS^12^. The activities of superoxide dismutase (SOD) and glutathione peroxidase (GPx) constitute a first-line antioxidant defense system that plays a key and fundamental role in the total defense mechanisms and strategies in biological systems^81^. Glutathione (GSH) is a well-known antioxidant scavenger for free radicals. The reduced form of glutathione plays a critical role in controlling cellular levels of ROS^82^. Animals treated with the VCN exhibited a marked improvement in the oxidative stress markers compared to the induced group; this can be explained by the effect of VCN on the enhanced activity of the antioxidant nuclear transcriptional factor Nrf2 (a transcription factor that regulates cell responses to oxidative stress and protects against oxidant injury) as was observed in the gene expression analysis of the lung tissue, and supported by a previous study demonstrating the effect of VCN on oxidative stress-induced hepatotoxicity in vitro and in vivo by stabilizing the Nrf2 and preventing its proteasomal degradation via inhibiting the binding of Keap1 and Nrf2^83^. Also, it has been documented that VCN effectively reduced raised hepatic MDA and NO levels and LDH activity and enhanced decreased hepatic GSH levels in a rat model of hepatic fibrosis^34^.

**Figure 9:**
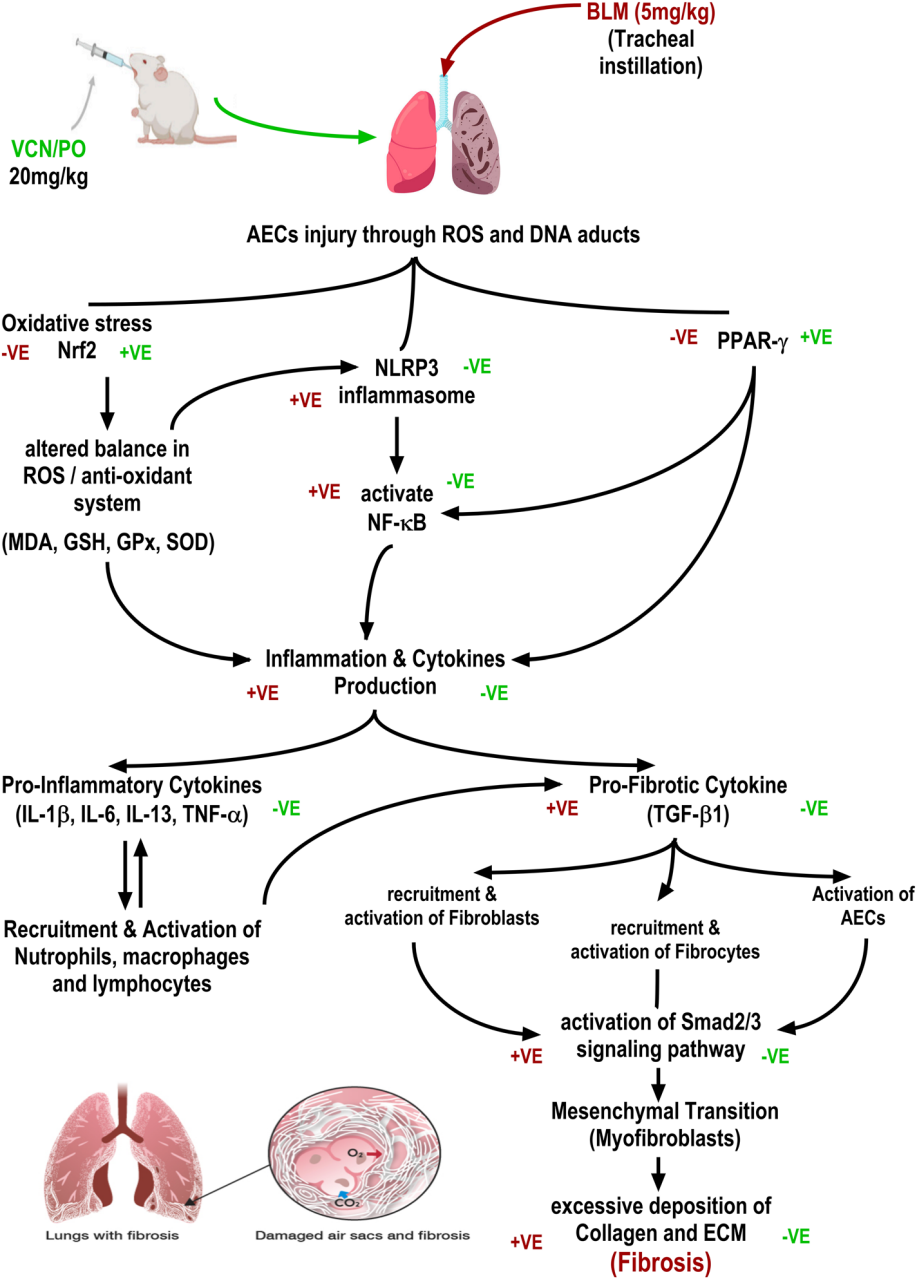
Schematic illustration summarizing the effect of vinpocetine (VCN) on the outcome of pulmonary fibrosis. The green symbol represents the effect of VCN, and the red symbol represents the effect of BLM.

To elucidate the most probable mechanism of action of the anti-fibrotic effect of VCN in the BLM-induced pulmonary fibrosis in vivo, the effect of VCN was tested in TGF-β1-induced fibrogenesis in the MCR-5 cell line. Results of cytotoxicity/viability revealed that VCN exhibited low cytotoxicity against normal human lung fibroblasts (MRC-5). Results of gene expression of (fibronectin, vimentin, α-SMA, and Smad2/3) in MCR-5 revealed that cells treated with TGF-β1 only (10 ng/ml) expressed high levels of the targeted genes, proving the fact that TGF-β1 is a fibrogenic cytokine and a key player and regulator in the development and progression of pulmonary fibrosis by promoting the activation, proliferation, and migration of fibroblasts, ECM deposition and the transformation from fibroblasts to myofibroblasts^84,85^. The transition from fibroblasts and other mesenchymal cells to myofibroblasts (MFBs) is critical to developing and progressing pulmonary fibrosis^86^. Where TGF-β1 activates fibroblasts through Smad2/3 signaling transduction, which results in increased expression of ECM components (fibronectin and vimentin) that further participate in the proliferation, migration, and trans-differentiation of fibroblasts into myofibroblasts that express a high level of α-SMA^65^, VCN managed to reduce the expression of the fibrotic indices for ECM deposition and the signaling protein (Smad2/3) involved in the activation of fibroblasts and transitioning into ECM-producing myofibroblasts in MRC-5 induced with TGF-β1. VCN produced its effect probably due to either blocking the binding of TGF-β1 to its specific receptors (TGF-βRI and TGF-βRII) or inhibiting the production and activation of the intracellular signaling protein (Smad2/3) or other indirect signaling pathways, thus suppressing the TGF-β1/Smad2/3 pathway necessary for the activation, proliferation, and fibroblast transition into myofibroblast and halting further production of collagen and other components of ECM (vimentin, fibronectin, and α-SMA). To support our claim regarding VCN, no literature states explicitly that there is a direct effect of VCN on certain fibrosis signaling pathways; however, previous studies done on liver fibrosis and cardiac fibrosis in vivo and in vitro demonstrated that VCN was able to reduce the accumulation of fibrotic components possibly through its main pharmacological mechanism by targeting the PDE1A, where the latter has been identified as a key regulator of fibroblast activation and ECM remodeling in the heart^87^. This isoform was specifically enhanced in activated myofibroblasts in vitro upon angiotensin II and TGF-β1 stimulation and in vivo in mouse, rat, and human fibrotic hearts^88^. Figure 9 summarizes the effects of vinpocetine on the outcome of pulmonary fibrosis.

The study recommends further investigations with different dosing frequencies of VCN, a combination of VCN, and PFD in different doses to evaluate the possible synergism or additive effects. The study also recommends studying the impact of VCN on other molecular targets like the apoptotic-related targets (Bcl-2, BAX, and caspase) and the NOX family related to ROS production. Finally, the study recommends a pilot clinical investigation to confirm the beneficial effects of VCN in patients with interstitial fibrosing disorders. The study recommends using different time-line modalities starting at day 10 or 14 of the induction to rule out the therapeutic benefits.

## Conclusion

Vinpocetine expressed a remarkable attenuating effect in both in vivo and in vitro, making it a candidate for further clinical investigation in cases of pulmonary fibrosis. VCN managed to halt the progression of fibrosis either directly by interfering with the classical TGF-β1/Smad_2/3_ signaling pathway or indirectly by upregulating the expression of Nrf2, enhancing the antioxidant system, activating PPAR-γ and downregulating the NLRP3/NF-κB pathway which influences the production of inflammatory cytokines involved in the pathogenesis of pulmonary fibrosis.

## Data Availability

Zenodo: The Mitigating Effect of Vinpocetine in Experimental Pulmonary Fibrosis [Data set]. https://zenodo.org/doi/10.5281/zenodo.10615183. The project contains the following underlying data: Data sets of The Mitigating Effect of Vinpocetine in Experimental Pulmonary Fibrosis. Data are available under the terms of the Creative Commons Attribution 4.0 International license (CC-BY 4.0).
